# A systematic study of emergency strategies for skin healing after pediatric burns: a comprehensive review and a multidisciplinary perspective

**DOI:** 10.1186/s13052-025-02066-9

**Published:** 2025-07-15

**Authors:** Luigi Coppola, Alessandra Cianflone, Pasquale Primo, Alessandra Macrì, Fiorenza Mastrodonato, Angela Iannicelli, Rosanna Parasole, Francesca Zamparelli, Marcello Zamparelli, Peppino Mirabelli

**Affiliations:** 1 UOS Laboratori di Ricerca e Biobanca, Santobono-Pausilipon Children’s Hospital, AORN, Naples, Italy; 2 UOC Clinical and Translational Research, Santobono-Pausilipon Children’s Hospital, AORN, Naples, Italy; 3 UOSD Plastic Surgery and Pediatric Burns Center, Santobono Pausilipon National Children’s Hospital, Naples, Italy

**Keywords:** Burns injury, Emergency, Pediatrics, ATMPs

## Abstract

Burn injuries are a significant cause of morbidity and mortality in pediatric populations. Our study aims to provide an in-depth overview of the current emergency treatment strategies for dermal injuries in children. While advances in burn care have led to improved outcomes for patients with dermal injuries, there remains considerable variability in treatment approaches, and numerous opportunities exist to enhance patient care through the adoption of novel therapies and more effective management of healthcare resources. Children with severe burns often experience profound physiological changes that can result in a range of complications, including infections, fluid and metabolism imbalance due to increased catabolism and protein requirements. Our systematic review focuses on emergency treatment approaches that have demonstrated direct application in pediatric burn care. To this aim, we consulted the scientific databases PubMed, Embase, Web of Science, and Cochrane Library, selecting *n* = 6 studies that outline the emergency therapeutic approach used in the pediatric setting after a burn, highlighting the most widely adopted techniques and their clinical applications. Study selection was guided by defined eligibility criteria; only original articles published in English that investigated emergency interventions in pediatric populations (0 to 17 years) were considered. Studies were excluded if they involved subjects over 17 years of age, focused on conditions unrelated to burns, employed in vitro or in vivo models, or were review articles. Through a comprehensive examination of these approaches, our study aims to identify areas for improvement and potential advancements in pediatric burn management, suggesting possible future approaches based on Advanced therapy medicinal products (ATMPs) storage approaches.

**Supplementary Information:**

The online version contains supplementary material available at 10.1186/s13052-025-02066-9.

## Background

Burn injuries are a significant cause of morbidity and mortality in pediatric populations [1]. Despite several advances to improve outcomes for patients with dermal injuries, considerable variability remains in treatment approaches, and numerous opportunities exist to enhance patient care through the adoption of novel and more effective therapies, improving the management of healthcare resources [2]. The treatment of children requires collaboration between multiple specialties, including pediatricians, plastic surgeons, nurses, physical therapists, psychologists, and pediatric intensive care specialists [3]. Each phase requires specific expertise to optimize outcomes and minimize complications [4]; the plastic and reconstructive surgery involves skin grafting [5], treatment involving the transplantation of autologous skin (from the same patient) [6] or, alternatively, the use of temporary allogenic skin grafts (such as pig skin or synthetic skin) to promote healing [7]. Actually, in most emergencies, surgical interventions or innovative dressing are employed to facilitate the restoration of compromised tissue states [8]. In borderline cases involving severe burns over large body surfaces, skin expansion is considered. In such cases, devices may be used to expand healthy skin and improve its use as a graft [9]. Children with severe burns often experience profound physiological changes that can result in a range of complications, including infections, fluid imbalance, and long-term issues related to growth and development [10, 11]. At our burn unit at the Azienda Ospedaliera Rilievo Nazionale (AORN) Santobono - Pausilipon (Naples, Italy), several emergency surgical interventions are performed, involving in extreme cases foreign facilities to obtain amplified skin biopsies; the production is certified Good Manufacturing Practices (GMPs) and guaranteed by the Wyss Zurich Translational Center (University of Zurich, Switzerland) as previously published [12]. The use of this approach is giving our hospital good results; however, it is essential to evaluate approaches currently used for pediatric burn patients, trying to identify innovative and ready-to-use strategies. Our study of the literature aimed to provide an in-depth overview of the current emergency therapeutics strategies used after burns in pediatric patients; indeed, it focuses on treatment approaches that have demonstrated direct application in pediatric burn care, which could provide an overview and important insights for future developments. Through a comprehensive examination of these approaches, our manuscript focused on identifying areas for improvement and potential advancements in pediatric burn management. In this scenario, we decided to carry out a systematic review associated with advanced therapies in skin regeneration associated with pediatric field. Our proposal was focused on the analysis of 4 different databases, allowing us to make in-depth evaluations of potential evolution of upcoming applications associated with burns pediatric patients at our hospital. We have described the methods adopted for the screening, eligibility, and inclusion that led us to the selection of the *n* = 6 studies, showing the interventions used in pediatric patients, and highlighting the most widely adopted techniques and their clinical applications. We hope that our critical and in-depth analysis will serve as a valuable resource to evaluate potential applications of innovative methodologies in the pediatric field, particularly in the context of burn management. Burn injuries represent a significant clinical emergency requiring immediate and effective intervention due to the unique physiological and psychological vulnerabilities of children. The development and implementation of advanced therapeutic strategies are key to improving outcomes, minimizing long-term complications and improving the quality of life of children. Therefore, the search for novel and evidence-based approaches in the treatment of pediatric burns is of paramount importance, emphasizing the need for continuous research and innovation in this critical area of medical science.

## Materials and methods

The paper was prepared based on the Preferred Reporting Items for Systematic Reviews and Meta-Analyses (PRISMA) standards and guidelines (See Supplementary Material 1) [13]. All of the data originates from approaches that are currently being published in peer-reviewed international journals.

### Search strategy and eligibility criteria

The terms “advanced therapy” and “skin regeneration” were used to search four databases such as Pubmed, Embase, Web of Science and Cochrane Library. For eligibility criteria on study characteristics, we included English peer-reviewed papers involving burn injuries pediatric patients. All types of articles were included, while reviews were excluded. All studies were identified by searching the PubMed, Embase, Web of Science, and Cochrane Library databases by using the following search keywords “advanced therapy” and “skin regeneration” with the last search date on November 2024.

### Data extraction and collection

The following criteria were applied for study selection, including the following inclusion criteria:

- emergency approaches adopted in the pediatric field,
- children aged 0–17 years,
- articles in English.

Regarding the exclusion criteria as mentioned above:

- review articles,
- age of participants > 17 years,
- the study of pathologies other than burns.
- *in vitro/vivo* approaches.

Two authors carried out article searches and data collection independently (L.C. and A.C.); a third reviewer (P.P.) independently carried out data extraction and reviewed the selected published articles to confirm that they met the inclusion criteria. Any disagreements that arose between the reviewers were resolved through discussion with a fourth and fifth reviewer (A.M., F.M., A.I.). Also, R.P., M.Z and P.M. supervised the activities and gave final approval to the study.

### Risk of bias

We performed the risk of bias assessment by objectively evaluating the selected publications with the following items: i) Is the clinical management adequately described? (ii) Is the therapeutic approach adequately described? (iii) the number of cases adequate to recommend the emergency approach? We assessed the risk of bias among studies using the Newcastle-Ottawa scale (NOS) for assessing study quality. We assigned 1 to 3 stars (*) for each items.

### Quality assessment of selected studies

We performed a quality assessment using Newcastle-Ottawa Scale (NOS) for the selected studies (See Supplementary material 2) [14]. We assigned scores for the selected studies regarding the description in the papers selected for the final review regarding (i) clinical management of the patient (ii) therapeutic approach adopted; (iii) number of cases assessed.

## Results

### Study selection

**Fig. 1:**
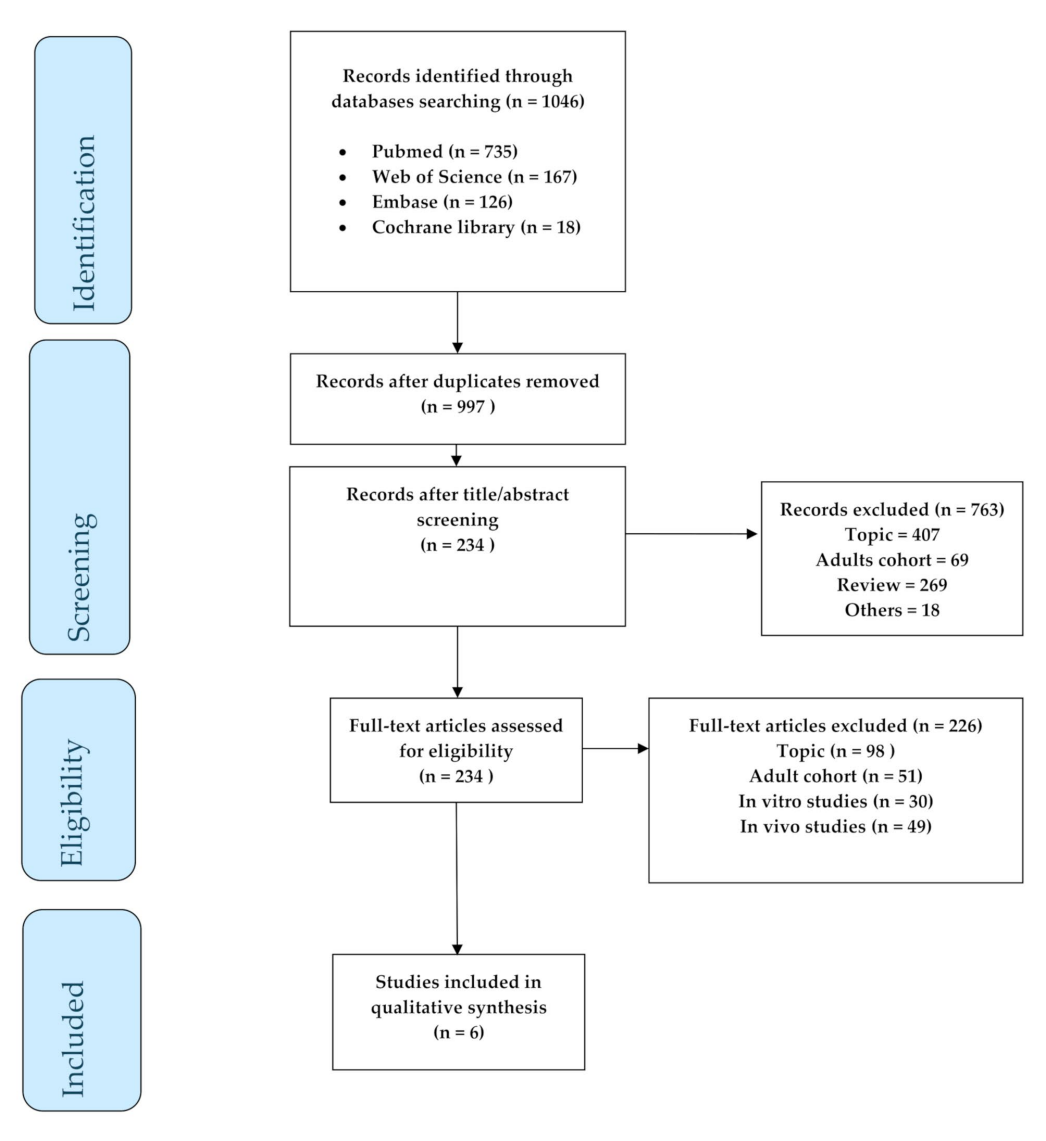
PRISMA flow diagram: flow diagram of the identification, screening, and inclusion of the *n* = 6 eligible studies according to the PRISMA statement

We included approaches directly performed on children according to the PRISMA flow diagram shown in Fig. 1. We selected a total of *n* = 1046 articles; in the first phase of screening, *n* = 49 duplicates were removed using Excel (Microsoft Office 2019, software), and a total of *n* = 997 articles were analyzed. Subsequently, *n* = 763 papers were excluded based on title and abstract. The rationale for exclusions is due to the topic being different from the focus of the original question (*n* = 407), to the exclusion of review articles (*n* = 269) or papers focused on an adult cohort (*n* = 69); additionally, *n* = 18 articles were excluded as they were not accessible to our queried database platforms. Subsequently, 234 full-text articles were assessed for eligibility; of the selected papers, *n* = 226 were excluded for the different topic covered different from burn injuries (*n* = 96), studies focused on adult subjects (*n* = 51 aged > 17 years). Also, in vitro (*n* = 30) and in vivo approaches (*n* = 49) that did not show direct application in children were excluded from the final analysis. Finally, we included *n* = 6 full-text articles for the final review.

### Study characteristics

A total of *n* = 6 studies were selected and included in the final qualitative synthesis. The main characteristics are listed in Table 1; all of the studies focus on treating a very small population, primarily due to they are involving children; as shown all of the studies used biomaterials to enable faster recovery from the burn. Today, the gold standard approach for burns treatment is autologous skin transplantation; among the selected papers, M. A. Noureldin et al. [15] have managed a randomized prospective comparative study of *n* = 40 pediatric burn patients with deep burns to evaluate the management of skin grafts with two different techniques [15]. In this study, patients were divided into two groups, “Meek” and “meshed” groups. The meshing technique remains the standard of care when skin autograft expansion is required; however, the modified meek micrografting technique represents a possible solution to the problem of limited donor sites and a large raw area to cover and also has the potential to provide a better cosmetic outcome. They used the Patient and Observer Scar Assessment (POSAS) scale, which used a series of parameters to assess wound recovery. The POSAS assessments showed better results for the Meek group with a mean score of 3.17 and for the mesh group, it was 4.2. The overall observer score was also better for the Meek group with a mean overall opinion score of 2.89 and for the mesh group, it was 4.1. The study establishes that the Meek technique is useful for covering burn wounds with a faster rate of expansion, more accessible application, better graft take-up and better scar appearance compared to the traditional mesher technique but it represents a longer time and more expensive approach. Unfortunately, autologous skin grafts are limited in availability and xenogeneic skin substitutes are emerging as potential innovative skin grafts. Skin substitutes from pigs or human cadavers may be exposed to autoimmune responses and infections and additional challenges include cultural and religious barriers of the subjects undergoing such treatments. In this context, Biazar et al. [16] involved the use of fish skin as a scaffold, which is emerging lately as a potentially very valuable approach due to its regenerative potential [16]. Fish skin has not been linked to autoimmune reactions and it is useful in cases where autologous skin grafts are not possible. In this specific case, an 8-year-old girl with traumatic wounds was treated with platelet-rich fibrin gel, growth factor-rich plasma gel, and acellular fish skin. Wound surface area measured at different time points after the initial injury revealed accelerated wound healing in the fish skin-treated wounds. Lukish et al. [17] studied TransCyte, a bioactive skin substitute consisting of a polymer membrane on a nylon mesh, thus reducing the associated pain, time required, and cost of care for patients [17]. The study was conducted on *n* = 40 children and designed to determine if the use of TransCyte on 20 of 40 children is safe, effective, and reduces hospital costs. The authors noted that the use of TransCyte as a biologic wound dressing is effective and increases healing compared to standard burn therapy. In 2019, G. Delli Santi et al. [18] studied the use of a new wound dressing based on the novel material Eiratex, composed of a biosynthetic cellulose network that mimics the nanostructure of collagen [18]. In this pilot study, *n* = 5 pediatric patients with burns were treated with the Eiratex wound dressing after enzymatic debridement using NexobridTM. They observed a mean healing time of 31.2 ± 3.5 days from the occurrence of the burn proposing new biomaterials that promote the wound healing process. A similar approach was adopted by G. Gravante et al. [19]; they tested the efficacy of hyalomatrix PA as a temporary dermal substitute to cover deep partial thickness burns after dermabrasion before skin grafting. From the third to the fifth day after hospitalization, dermabrasion was performed on deep burned areas, which were covered with hyalomatrix PA [19]. They performed dressing changes every 7 days including *n* = 300 patients in the study; they observed that 83% of patients healed within 21 days proposing the combination of dermabrasion with a temporary dermal substitute as a feasible treatment for deep burns. In 2021, an interesting study was proposed by the Lausanne Burn Center, a center with significant experience in the management of pediatric burns; K. Al-Dourobi et al. [20] described a retrospective case-control study comparing two methods; they studied biological progenitor bandages versus Aquacel Ag, applied 10–12 days after injury. Biological progenitor bandages contain cultured and viable allogeneic fetal dermal progenitor fibroblasts and can promote the repair and regeneration of functional skin tissues by releasing cytokines and growth factors, potentially eliminating the need for subsequent skin grafts, reducing the formation of hypertrophic scar tissue. They did not observe any differences between groups in terms of length of hospitalization or initial rates of relative burn surface reduction. However, in the group treated with biological progenitor bandages, they observed differences in terms of hypertrophic scars and complications, and related corrective interventions [20]. Furthermore, since the Lausanne Burn Center has a very broad experience in the field they have proposed not only the ATMP production workflow but also the regulations for the use of this approach in clinical practice. They describe how to store and reuse the ATMP products when necessary for specific cases, reintroducing the concept of biobanking that is fundamental activating a regenerative medicine facility [21]. It proposes a useful approach to lay the foundations for activating a facility for the production and application of ATMP products in the clinical setting, spreading knowledge in this area and promoting the possible introduction in pediatric hospital facilities.

**Table 1:** Summary of the findings of the *n* = 6 eligible articles; the table provides a summary of the main characteristics of the manuscripts included in the final synthesis.

Authors	Journal	IF	Year	Methods used	Biomaterial	Size	Aim	Innovation	Findings	Doi
J. R. Lukish et al.	Journal of Pediatric Surgery	2.4	2001	Dressing changes and Transcyte dressing	TransCyte	40	Compare dressing change vs. TransCyte	New approach to closing wounds faster	TransCyte reduced the hospitalization time	10.1053/jpsu.2001.25678
G. Gravante et al.	Journal of Burn Care & Research	1.5	2007	Advanced Biopolymers as bridge treatment	Hyalomatrix	300	Remove necrotic debris	Create a temporary barrier function avoiding vapor loss and reducing bacterial invasion	Hyalomatrix generates easier and faster wound closure, improving aesthetic results	10.1097/BCR.0B013E318031A236
G. Delli Santi et al.	Burn open	3.2	2019	Wound dressing based	Eiratex and Nexobrid	5	Evaluate a new wound dressing biomaterial	Introduction of new wound dressing biomaterial	Epiprotect is a promising product for wound healing	10.1016/j.burnso.2019.05.001
K. Al-Dourobi et al.	Pharmaceuticals	4.3	2021	Advanced Therapy Medicinal Products, containing viable cultured allogeneic fetal dermal progenitor fibroblasts	Progenitor Biological Bandage and Aquacel	226	Study the effect of Progenitor Bandage and Aquacel on pediatric wound healing	Promote development of a ATMP products and evaluate two different dressing	Promoting a solution for early coverage of pediatric burn wounds	10.3390/ph14030201
M.A. Noureldin et al.	Burns	3.2	2022	Mesher and Meek technique	Skin grafting	40	Compare the mesher and the Meek technique	Emphasise the necessity of having the maximal benefit of the limited donor area	The Meek micrografting was found to be better when compared to the mesher	10.1016/j.burns.2022.01.016
E. Biazar et al.	Archives of Academic Emergency Medicine	5.4	2024	The gel-like application	Acellular Fish Skin as a scaffold	1	Apply aninnovative biomaterials	Fish skin as new wound healing application	The growth factor with acellular fish skin graft were effective in healing traumatic wounds	10.22037/aaem.v12i1.2103

### Burns prevention in the pediatric field

Burns causes long-term physical, psychological and socioeconomic outcomes; their incidence is higher in low-income countries due to reduced safety measures and training [22]. Pediatric burn prevention is critically dependent on structured education, parental awareness, and the implementation of evidence-based strategies. Primary prevention requires that caregivers adopt safety measures such as restricting access to hot liquids, electrical devices, and open flames, while also instructing children on risk-avoidant behaviors [22, 23]. Also, equally vital is knowledge of appropriate post-burn first aid, including immediate cooling and prompt medical evaluation, to mitigate complications. In this context, the pediatric hospitals will increase their dual role in both clinical management and prevention through structured educational interventions, visual materials, and community outreach campaigns aimed at reducing incidence and enhancing caregiver competence [24].

## Discussion

It is essential to underline that the current preventive strategies in Italy and in the world are not sufficient to contain and reduce burns in pediatric field. This is due to inadequate training and habits that do not reduce the risk. It would be fundamental to implement such as pictures, postcards, or even basic usable recommendations in the Hospitals and via websites or app with educational purposes. At AORN Santobono-Pausilipon, there are illustrative educational pictures for children and parents. In addition, workshops and informative seminars are periodically organized to reduce the risk of children’s exposure to burns. Regarding additional medical centers worldwide, there are several examples to follow, such as the Royal Children’s Hospital in Melbourne (Australia, https://www.rch.org.au/clinicalguide/guideline_index/burns/) [25] or, alternatively, Johns Hopkins Medicine in Baltimore (USA, https://www.hopkinsmedicine.org/health/conditions-and-diseases/burns/burns-in-children) to implement awareness and promote a mitigation of such events. In this scenario, our review has proposed a current vision of the real approaches carried out in the pediatric field. As it is known, burns require fast and targeted interventions and in this sense applying stable and clear emergency protocols could make the difference between life and death for children. We have selected and described in the final review *n* = 6 papers, describing the approaches used and the results obtained by the research groups. It is important to underline that in the screening phase of papers were excluded, although preliminary and not of interest for our final review, proposed encouraging results on animal models, mostly mice and in a smaller number of cases pigs or others. Several preclinical approaches involved the use of biopolymer matrices of various natures, nanogels, or alternatively biophysical approaches, but although considered interesting, they were not protocols ready for use on humans. Regarding ready-to-use approaches in a pediatric burn emergency, they are mainly based on innovative surgical approaches, cutting-edge dressings, and innovative approaches that provide the use of ATMP products. We consider the study by Karim Al-Dourobi et al. particularly interesting [20]; indeed, they presented not only an innovative approach for burn regeneration in children but also presented a very detailed description of the Lausanne Burn Center in the production of ATMP, specifically progenitor biological bandages. In fact, they described the FE002-SK2 cell line technical characteristics and related GMPs. Furthermore, their study describes the importance of a biobank unit that preserves biomaterial ready for use in emergency scenarios for pediatrics burns. Regarding ATMPs, they could improve functional and aesthetic outcomes, speed recovery, and reduce long-term problems. Furthermore, these treatments have the potential to advance personalized medicine by enabling more specialized and efficient burn care, promoting the use of safe, quality, off-the-shelf products. Although our systematic review yielded interesting and valuable insights, it is essential to acknowledge several limitations that may affect the interpretation of our results. First, despite a comprehensive search of four major databases, the results are inherently constrained by the keywords and search terms we selected. Although these terms were carefully chosen to align with the scope of our study, there is a possibility that the use of alternative keywords or search strategies, either by industry experts or professional librarians, may have yielded a different set of studies, potentially skewing the review results. Additionally, our review only included studies published in the selected databases, which may have introduced bias. Research published in non-indexed or less accessible journals, or those that are not available in English, may have been excluded, limiting the generalizability of our results. Another limitation is the potential for selection bias within individual studies. Many of the studies included in the review may have had small sample sizes, lack of control groups, or other methodological shortcomings that could impact the strength of the conclusions drawn. Despite our efforts to include only high-quality studies, limitations of the primary research studies themselves cannot be completely eliminated by our review process. Finally, it is important to note that our review focuses primarily on available data, which, in some cases, may be outdated or limited to specific geographic regions or healthcare settings. Given these limitations, although our review provides valuable insights into current strategies for skin healing in pediatric burn patients, additional research using broader and more diverse methodologies, updated data, and a broader range of search parameters is needed to refine and extend the findings presented here. An increased focus on clinical research on pediatric burn injuries is of paramount importance for the development of innovative and more effective treatment strategies, with the ultimate goal of improving the overall prognosis and quality of life of children. Given the challenges faced by pediatric patients, a deeper understanding of the mechanisms underlying burn injuries in children is crucial for the identification of age-specific therapeutic interventions. New medical research in this field will not only facilitate the discovery of new pharmacological agents and advanced wound care techniques but will also allow existing recovery protocols to be refined and adapted to the specific needs of young patients. In addition, new research approaches could improve infection control practices and promote faster and more efficient healing processes. Finally, a sustained and focused approach to pediatric burn research will significantly contribute to minimizing the long-term physical and psychological consequences commonly associated with burn injuries, such as scarring, functional disability, and post-traumatic stress disorder.

## Conclusions

 [26]The international guidelines such as the American Burn Association (ABA) [26], the European Burns Association (EBA) [27], and the International Society for Burn Injuries (ISBI) [28] provide recommendations for the pediatric burn patients management and treatments. These recommendations are useful to develop standardized care protocols and enhancing results in burn events involving children. Despite these international guidelines, clinical practice requires a further increase towards standardization, and in this scenario, our study highlights the medical need to consolidate effective practices through the identification of key areas of development, such as ATMPs, to provide innovative organizational models and advanced therapeutic strategies.

## Electronic supplementary material

Below is the link to the electronic supplementary material.

Supplementary Material 1

Supplementary Material 2

## Data Availability

The data that support the findings of this study are available from the corresponding author upon reasonable request.

## Abbreviations

AORN: Azienda Ospedaliera di Rilievo Nazionale
ATMPs: Advanced therapy medicinal products
ABA: American Burn Association
EBA: The European Burns Association
GMP: Good Manufacturing Practice
NOS: Newcastle Ottawa Scale
ISBI: International Society for Burn Injuries
POSAS: Patient and Observer Scar Assessment
PRISMA: Preferred Reporting Items for Systematic Reviews and Meta-Analyses
