# Supplementary material for: A systematic study of emergency strategies for skin healing after pediatric burns: a comprehensive review and a multidisciplinary perspective
Source: Ital J Pediatr. 2025 Jul 15;51:233. doi: 10.1186/s13052-025-02066-9 (PMC12261643; doi:10.1186/s13052-025-02066-9)
Supplement: Supplementary file 2 — Supplementary Material 2 [file 13052_2025_2066_MOESM2_ESM.docx]

**Supplementary material 2:** Newcastle - Ottawa quality assessment Scale of selected studies of our final revision. A study can be awarded a maximum of one star for each numbered items. A maximum of three stars can be given for each papers.

| **Authors** | **Year** | **Item 1** | **Item 2** | **Item 3** | **Score** |
| --- | --- | --- | --- | --- | --- |
| E. Biazar et al. | 2024 | ** | *** | * | 6 |
| M.A. Noureldin et al. | 2022 | ** | *** | *** | 8 |
| J. R. Lukish et al. | 2001 | *** | *** | *** | 9 |
| G. Delli Santi et al. | 2019 | *** | ** | ** | 7 |
| G. Gravante et al. | 2007 | * | *** | *** | 7 |
| K. Al-Dourobi et al. | 2021 | *** | *** | *** | 9 |

**CODING MANUAL**

- **Item 1: Is the clinical management adequately described?**

* Star: The description of clinical management is basic, with significant gaps in essential information. Components are missing, and the details provided lack clarity and precision.

** Stars: Clinical management is well described, but several details are underdeveloped or presented in an ambiguous manner. While the overall understanding is feasible, further clarification and elaboration could have been helpful to improve completeness.

*** Stars: Clinical management is comprehensively described, with all critical components covered in depth. The explanation is precise, clear, and well-organized, providing a thorough understanding of the topic.

- **Item 2: Is the therapeutic approach adequately described?**

* Star: The therapeutic approach is not adequately described, with significant gaps or a lack of important details.

** Stars: The therapeutic approach is described, but certain aspects remain vague or underexplored.

*** Stars: Yes, the therapeutic approach is thoroughly and clearly described, with all necessary details provided.

- **Item 3: Is the number of cases adequate to recommend the emergency approach?**

* Star: The number of cases is not sufficiently representative to make a reliable recommendation for the emergency approach

** Stars: The number of cases is somewhat adequate, but additional data would strengthen the recommendation for the emergency approach.

*** Stars: Yes, the number of cases is sufficiently large and diverse to confidently recommend the emergency approach.
